# Differential Effects of E-Cigarette on Microvascular Endothelial Function, Arterial Stiffness and Oxidative Stress: A Randomized Crossover Trial

**DOI:** 10.1038/s41598-018-28723-0

**Published:** 2018-07-10

**Authors:** Martin Chaumont, Benjamin de Becker, Wael Zaher, Antoine Culié, Guillaume Deprez, Christian Mélot, Florence Reyé, Pierre Van Antwerpen, Cédric Delporte, Nadia Debbas, Karim Zouaoui Boudjeltia, Philippe van de Borne

**Affiliations:** 1 Department of Cardiology, Erasme University Hospital, Université Libre de Bruxelles, Brussels, Belgium; 2 Institute for Translational Research in Cardiovascular and Respiratory Sciences, Université Libre de Bruxelles, Brussels, Belgium; 3 Department of Clinical Chemistry, Université Libre de Bruxelles, Brussels, Belgium; 4 Laboratory of Pharmaceutical Chemistry, Faculty of Pharmacy, Université Libre de Bruxelles, Brussels, Belgium; 5 Department of Cardiology, Saint-Pierre Hospital, Université Libre de Bruxelles, Brussels, Belgium; 6 Laboratory of Experimental Medicine (ULB 222 Unit), CHU de Charleroi, A. Vésale Hospital, Université Libre de Bruxelles, Montigny-le-Tilleul, Belgium

## Abstract

Propylene glycol and glycerol are electronic cigarettes vehicles allowing liquid vaporization and nicotine transport. The respective effects of these different constituents on the cardiovascular system are unknown. We assessed the differential effects of vehicles (propylene glycol and glycerol) and nicotine on microcirculatory function, arterial stiffness, hemodynamic parameters and oxidative stress. Twenty-five tobacco smokers were exposed to vaping with and without nicotine, and sham vaping, in a randomized, single blind, 3-period crossover design study. Neither sham-vaping nor vaping in the absence of nicotine resulted in modifications of cardiovascular parameters or oxidative stress. In contrast, vaping with nicotine: 1) impaired acetylcholine mediated vasodilation (mean ± standard error mean) (area under curve, perfusion unit (PU), 3385 ± 27PU to 2271 ± 27PU, *p* < *0.0001*); 2) increased indices of arterial stiffness, namely augmentation index corrected for heart rhythm (−3.5 ± 1.5% to 1.9 ± 2.3%; *p* = *0.013*) and pulse wave velocity (4.9 ± 0.1 m.s^−1^ to 5.3 ± 0^.1^ m.s^−1^; *p* < *0.0001*); 3) increased systolic and diastolic blood pressures as well as heart rate (all *p* < *0.0001*) and finally; 4) raised plasma myeloperoxidase (median [interquartile range]) (13.6 ng.ml^−1^ [10–17.7] to 18.9 ng.ml^−1^ [12.2–54.4], *p* = *0.005*). Our findings demonstrated that high temperature e-cigarette vehicle vaporization does not alter micro- and macro-vascular function, and oxidative stress, and that these effects are solely attributable to nicotine.

## Introduction

Electronic cigarettes (e-cigarettes) are battery-powered devices that aerosolize a liquid (i.e. “e-liquid”) composed of propylene glycol (PG) and/or glycerol (GLY), flavorings and most commonly, nicotine. Suspension of fine particles in a gas forms the aerosol, that is inhaled (i.e. “vaped”)^1,2^. Literature suggests that e-cigarettes vaping may be safer than tobacco smoking^3,4^. In addition to be a smoking cessation aid^5^; e-cigarettes are also used for recreational purposes^6^. About 5% of adults in the western world are current e-cigarettes users^7^.

Since its first sales in the 2000s, the growing e-cigarettes market has quickly evolved and is poised to grow over $47 billion by 2025^8^. E-cigarettes are becoming increasingly powerful and deliver now high energy level to low coil resistance (sub-ohm vaping). Sub-ohm vaping increases the temperature of the coil in contact with e-liquid impregnated wick^9^. The sub-ohm modality enhances vapor and heat production^9–12^, and is accompanied by a decrease of nicotine concentration in the e-liquid, which is in turn vaped in larger amount to maintain a sufficient nicotine plasma level^13,14^.

Several studies have demonstrated endothelial dysfunction, oxidative stress imbalance and arterial stiffness increase after vaping e-cigarette with nicotine^15–17^. The pharmacological actions of nicotine make it impossible to distinguish the respective effects of the carriers themselves (PG and GLY) and nicotine on the endothelial dysfunction, oxidative stress and arterial stiffness increase they observed^15^. Nicotine is an alkaloid which rise blood pressure and heart rate, and induces vasoconstriction through mechanisms such as catecholamine releases and endothelial dysfunction^18^. PG and GLY can undergo combustion when vaporized at high wattage and thereby produce carbonyls, which are known cardiovascular toxicants^15,19,20^. Although the carbonyls produced in realistic vaping conditions are likely far less than during tobacco combustion^21^, a potential toxic effect on the cardiovascular system cannot be excluded^15^.

Using a placebo-controlled, randomized, single blind crossover design study, we tested the hypothesis that the PG and GLY carriers do not affect the cardiovascular system in contrast to the nicotine contained in the e-liquid which exerts toxic effects on micro- and macrovascular function when occasional tobacco smokers vape at high temperature.

## Methods

Additional information on the study design and the details of the methods is provided in the data supplement. The datasets generated during and/or analyzed during the current study are available from the corresponding author.

### Participants

Twenty-five healthy occasional tobacco smokers (18 males; mean age 23 ± 0.4 years; weight 72 ± 2 kg; height 178 ± 2 cm; body mass index 23 ± 0.4 kg.m^−2^; median cumulative pack-year 0.2 [interquartile range 0.1–0.8]) were enrolled at our academic center (Erasme University Hospital, Brussels, Belgium) between January 2017 and November 2017. Participants were enrolled on the basis of their excellent vaping tolerance. An informed consent was obtained from all the study participants after having received complete information about the study design, which was approved by the local Ethics Committee (Hôpital Erasme - CCB: B406201629930) and conformed to the Declaration of Helsinki. The study is registered at ClinicalTrials.gov (January 30, 2017), identifier NCT03036644.

### Study design, randomization and masking

This randomized study was placebo-controlled, single-blind with a three-period crossover design. The periods consisted of: 1) vaping without nicotine; 2) vaping with nicotine; and 3) sham-vaping. Allocation to the sequence order was performed according to a computer-generated randomization list (Flow diagram of the participants course during the study - CONSORT–in appendix). The investigators were unaware of the experimental session since allocation and exposure to the sessions was supervised by an unblinded member who did not participate in any other aspect of the study. Volunteers were unmasked since they could notice that the vaping device was turned on or off.

### Vaping protocol

The carrier used in the two e-liquids was a mix of 50% PG and 50% GLY pharmaceutical grade (Fagron^©^, Waregem, Belgium). One e-liquid was nicotine free (0 mg.ml^−1^), whereas nicotine (Nicobrand^©^, Coleraine, UK) was added to the other one at a concentration of 3 mg.ml^−1^. A last-generation high-power vaping device with popular and commercially available parts in U.S (Smoke^©^, Shenzen, China) was used. E-cigarettes were set-up at 60 Watts (0.4Ω dual coils). Vaping sessions (with and without nicotine) consisted of 25 puffs (4-s puffs at 30-s intervals) in order to create sub-ohm vaping conditions (personal data, please see data supplement)^9^. During the sham-vaping session, strict supervision of the participants ensured that they followed exactly the same respiratory maneuvers, but with the e-cigarette turned off.

### Study assessments

#### Indices of skin microcirculatory blood flow (Laser Doppler imager (Moor^©^))

By using a laser Doppler imager (MOORLDI2-IR, Moor Instruments^©^, Axminster, UK)^22^, we measured acetylcholine (Ach) and sodium nitroprusside (SNP) hyperemia at the level of the right forearm (middle part) 60 minutes after vaping and sham-vaping. Ach and SNP were administered percutaneously using iontophoresis^22,23^. On the contralateral forearm, we tested the skin hyperemic response to local heating after pretreatment with L-*N*-arginine-methyl-ester (L-NAME) or saline (placebo) iontophoresis, to evaluate nitric oxide (NO)-mediated vasodilation^22,23^. Skin blood flow was measured automatically (LDI version 5.3D software, Moor Instruments^©^) and was expressed in perfusion units, PU (arbitrary units of blood flow).

#### Hemodynamic parameters

Finger systolic and diastolic blood pressure (SBP and DBP) waveforms were obtained throughout the sessions with a beat-to-beat hemodynamic monitoring system (Finometer Pro, FMS^©^, Amsterdam, the Netherlands) on the right middle finger^24^.

#### Arterial stiffness assessment

Aortic wave reflection assessment: Central aortic hemodynamics, and augmentation index corrected for heart rate (AIx75), were estimated using pulse wave and wave separation analysis (SphygmoCor Px system^©^; Atcor Medical, Sydney, Australia) as previously described^25^. Measurements were made ten minutes before and five-minute after exposure.

Pulse wave velocity measurements: The carotid-femoral pulse wave velocity (PWV) was determined, immediately before and ten-minute after exposure, from sequential waveform measurements at carotid and femoral sites, by means of applanation tonometry and SphygmoCor software^26^.

#### Oxidative stress and nicotine assessment

Blood was drawn in the left antecubital vein 15 minutes before and 30 minutes after vaping (with and without nicotine) or sham-vaping. Total myeloperoxidase, protein-bound 3-chlorotyrosine and homocitrulline were measured in the plasma as previously described^27,28^. Nicotine was assessed in the serum before and 30 minutes after the nicotine vaping exposure by means of a mass spectrometer (Agilent QQQ 6490, Agilent^©^, Santa Clara, USA) with a jet stream electrospray ion source and an Agilent 1260 series LC system was used for quantification of plasma nicotine.

### Data analysis

This is presented in the Data Supplement.

### Outcome measures

The primary outcome was the impact of vaping on skin microcirculatory blood flow function (Ach mediated vasodilation). Secondary outcomes included continuous hemodynamic parameters, arterial stiffness and oxidative stress analyses after exposure.

### Statistical analysis

Continuous data were tested for normality using the Kolmogorov-Smirnov test. If the data were normally distributed, the data were expressed in mean ± (standard error of the mean) or if not median [interquartile range, P_25_–P_75_]. To our knowledge, the effects of vaping on skin microcirculatory blood flow has not yet been assessed. Therefore, sample calculation was not possible, but a posteriori computation leaded to more than 90% power for the primary end-point (Ach mediated vasodilation). We finally computed statistics on 21 subjects (7 by group, due to missing values in some groups) in a 3 × 3 cross-over trial testing three periods: vaping without nicotine, vaping with nicotine and sham-vaping in a random order. The statistical analysis consisted in 3 × 3 ANOVA for cross-over design. Carry-over effect and time effect were not significant. Therefore, the three periods were pooled leading to 21 measures for vaping without nicotine, vaping with nicotine and sham-vaping. The three periods were compared using an ANOVA for repeated measures or a Friedman test depending if the data were Gaussian or not. If the F-value was significant, pairwise comparisons were made using either a paired Student t-test or Wilcoxon signed rank test accordingly. The Bonferroni’s correction was applied. Effect of the three experimental sessions on hemodynamic variables were assessed by a two-factors (session, time) ANOVA with repeated measures on time and interaction session × time including baseline measurements. Pairwise comparisons were performed as previously described. Correlation analyses used the Spearman non-parametric correlation coefficient. All these analyses were performed using SPSS software version 22 (Chicago, USA). A *p-value* < *0.05* was considered significant.

### Data availability statement

The datasets generated during and/or analyzed during the current study are available from the corresponding author on reasonable request.

### As mentioned in the methods

An informed consent was obtained from all the study participants after having received complete information about the study design, which was approved by the local Ethics Committee (Hôpital Erasme - CCB: B406201629930) and conformed to the Declaration of Helsinki. The study is registered at ClinicalTrials.gov (January 30, 2017), identifier NCT03036644.

## Results

There were no differences in any baseline variables in the study population between the three sessions (Table 1). The mean e-liquid volume consumed after sham-vaping, vaping without nicotine and vaping with nicotine was 0.08 ± 0.02 ml, 2.1 ± 0.2 ml (*p vs*. sham-vaping < *0.0001*) and 1.7 ± 0.2 ml (*p vs*. sham-vaping < *0.0001*; and *p vs*. vaping without nicotine = *0.09*), respectively.

**Table 1:** Baseline parameters

	Vaping w/o nicotine	Vaping with nicotine	Sham-vaping	*p*
**Hemodynamic parameters**				
Heart rate (b.p.m)^*^	60 ± 2	59 ± 2	60 ± 2	>*0.7*
Humeral systolic blood pressure (mm Hg)^*^	110 ± 2	109 ± 1	110 ± 2	>*0.8*
Humeral diastolic blood pressure (mm Hg)^*^	68 ± 2	68 ± 1	68 ± 1	>*0.9*
**Arterial stiffness indices**				
Aortic sytolic blood pressure (mm Hg)^*^	95 ± 2	94 ± 1	94 ± 2	>*0.8*
Aortic diastolic blood pressure (mm Hg)^*^	69 ± 1	69 ± 1	68 ± 1	>*0.6*
Aortic pulse pressure (mm Hg)^*^	26 ± 1	26 ± 1	26 ± 1	>*0.9*
AIx75 (%)^*^	−4,5 ± 1.9	−3.5 ± 1.5	−3,4 ± 2.1	>*0.6*
Carotid–femoral PWV (m/s)^*^	4.9 ± 0.1	4.9 ± 0.1	5 ± 0.1	>*0.6*
SEVR^*^	184 ± 8	193 ± 7	184 ± 8	>*0.3*
**Oxidative stress biomarkers**				
MPO antigen (ng.mL^−1^)^†^	11.9 [3.6–165.5]	12.2 [4.2–81.1]	13.6 [7.9–26]	>*0.3*
P-B-3-Cl-Tyr/Tyr ratio (10^−6^)^†^	59.8 [49–67.5]	55.7 [47.8–64.1]	60.3 [48.2–71.9]	>*0.8*
Hcit/Lys ratio (10^−6^)^†^	218 [192.8–277.4]	230.1 [195.5–316.6]	222.3 [203–267]	>*0.8*

w/o, without; AIx75, Augmentation index corrected for heart rate; SEVR, subendocardial viability ratio; MPO, Myeloperoxydase; P-B-3-Cl-Tyr/Tyr, Protein-Bound-3-Chloro-thyrosine/Tyrosine ratio; Hcit/Lys: Homocitrulline/Lysine ratio; humeral systolic and diastolic blood pressure were assessed using auscultatory sphygmomanometer.

^*^Mean ± SEM.

^†^Median [interquantile range].

### Primary outcome

#### Effect of vaping on acetylcholine and sodium nitroprusside hyperemia (endothelial-dependent and -independent vasodilation)

**Figure 1:**
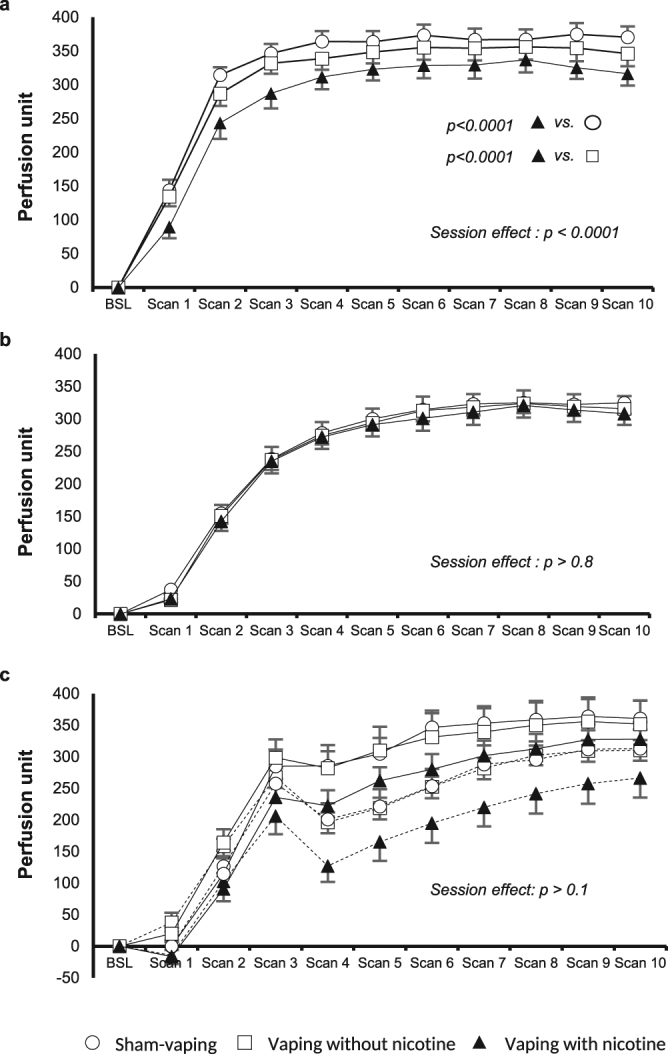
Endothelial microcirculatory function. Effect of (**a**) acetylcholine and (**b**) sodium nitroprusside iontophoresis after sham-vaping (open circle), nicotine free vaping (open square) and nicotine vaping (closed triangle). Effect of skin pretreatment by saline (continuous line) and L-N-arginine-methyl-ester (dotted line) iontophoresis on skin hyperemic response to local heating (**c**) after sham vaping, nicotine free vaping and nicotine vaping. Nicotine vaping decreased acetylcholine mediated vasodilation in comparison to sham-vaping (*p* < *0.0001*) and vaping without nicotine (*p* < *0.0001*). Sodium nitroprusside induced vasodilation was not affected by any of the three experimental sessions (ANOVA session effect; *p* > *0.8*). Vasodilation induced by heat in presence of saline or L-N-arginine-methyl-ester were not affected by vaping with and without nicotine and sham-vaping (ANOVA session effect; *p* > *0.1*). BSL indicates baseline; Scan 1 → 10 denotes the time from the first scan to the last scan (150 s per scan).

Sixty minutes after vaping with nicotine, Ach mediated vasodilation was decreased in comparison to sham-vaping (area under the curve (AUC), 3385 ± 27 PU to 2271 ± 27 PU; *p* < *0.0001*) and vaping without nicotine (AUC, 3207 ± 35 PU to 2271 ± 27 PU; *p* < *0.0001*) (Fig. 1a). Vasodilatory responses to SNP were not affected by nicotine vaping (*ANOVA* session effect; *p* > *0.8*) (Fig. 1b). Compared to vaping without nicotine or sham vaping, the Ach/SNP ratio decreased in case of nicotinic vaping from 1.3 ± 0.06 to 0.9 ± 0.05 (*p* < *0.001 vs* vaping without nicotine and *p* < 0.0001 *vs* sham-vaping). In comparison to sham-vaping, nicotine free vaping exposure did not affect skin vasodilator responses to Ach (*p* > *0.2*; Fig. 1a) nor SNP (*ANOVA* session effect; *p* > *0.8*; Fig. 1b).

#### Effect of vaping on skin thermal hyperemia in presence of L-*N*-arginine-methyl-ester (endothelial NO bioavailibility)

None of the three experimental sessions affected the skin vasodilatory response to heating in the control condition (pretreatment with normal saline) (*ANOVA* session effect; *p* > *0.9*; Fig. 1c) nor the response to heating after pretreatment with L-NAME (*ANOVA* session effect; *p* > *0.1)* (Fig. 1c) or the delta heating-AUC between control and L-NAME pretreated skin (*ANOVA* session effect; *p* > *0.1*).

### Secondary outcomes

#### Hemodynamic variables

**Figure 2:**
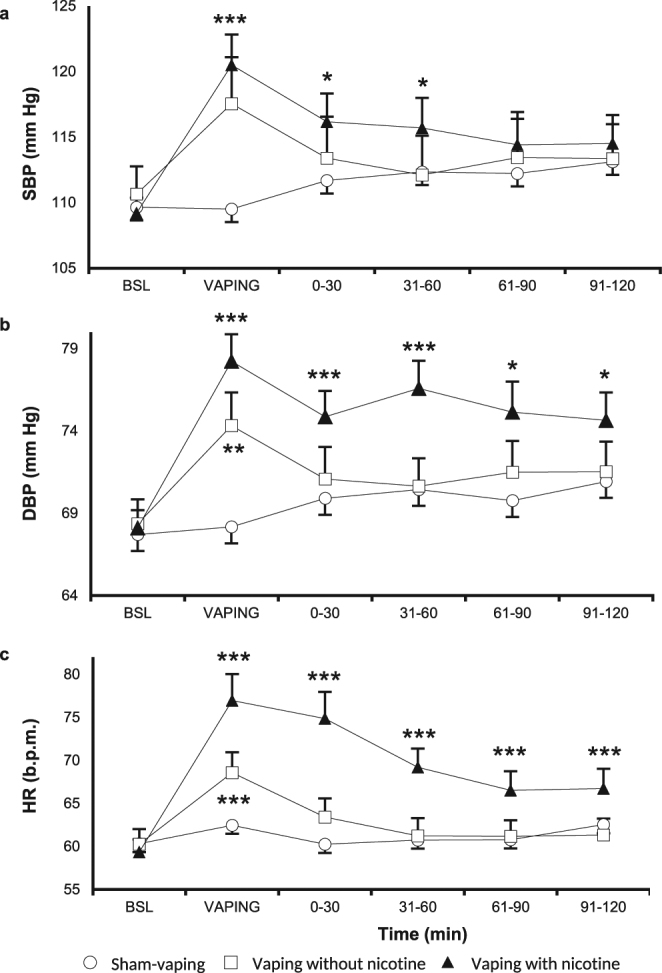
Hemodynamic parameters. Changes in (**a**) systolic blood pressure (SBP), (**b**) diastolic blood pressure (DBP) and (**C**) heart rate (HR) over time in the sham-vaping (open circle), nicotine free vaping (open square) and nicotine vaping (closed triangle) sessions. Nicotine vaping, compared to baseline (BSL), was associated with an increase in systolic and diastolic blood pressure as well as heart rate for 60 minutes, 120 minutes and 120 minutes post-exposure, respectively. In comparison to BSL, vaping without nicotine increased diastolic blood pressure and heart rate during the limited period of exposure. p-value for the time*session interaction effects for systolic and diastolic blood pressure as well as for heart rate were <*0.001*, <*0.0001* and <*0.0001*, respectively. p-value for comparisons vs. BSL: *p < *0.05*, **p < *0.01*, ***p < *0.001*. Data are the mean ± SEM.

In comparison to baseline, nicotinic vaping induced a sustained rise in SBP and DBP as well as in heart rate (Fig. 2) with the peaks reached during the exposure: 1) SBP from: 109 ± 1 mm Hg to 121 ± 2 mm Hg (*p* < *0.0001*; Fig. 2a); 2) DBP from: 68 ± 1 mm Hg to 78 ± 2 mm Hg (*p* < *0.0001*; Fig. 2b); and 3) heart rate from: 59 ± 1 bpm to 77 ± 3 bpm (*p* < *0.0001*; Fig. 2c). This rise in SBP persisted approximately 60 min after vaping, and lasted for 120 min for DBP and heart rate. Compared to baseline, nicotine free vaping increased SBP and DBP during the limited period of exposure but not thereafter: 1) SBP from: 111 ± 2 mm Hg to 118 ± 5 mm Hg (*p* < *0.001*; Fig. 2a); and 2) DBP from: 68 ± 1 mm Hg to 73 ± 2 mm Hg (*p* < *0.01*; Fig. 2b).

#### Aortic wave reflection and pulse wave velocity

In comparison to baseline, vaping with nicotine increased the AIx75 (−3.5 ± 1.5% to 1.9 ± 2.3%; *p* = *0.013*) and PWV (4.9 ± 0.1 m. s^−1^ to 5.3 ± 0.1 m. s^−1^; *p* < *0.0001*). In contrast, vaping without nicotine and sham-vaping affected neither AIx75 (*p* > *0.6 vs* baseline and *p* > *0.3 vs* baseline, respectively) nor PWV (*p* > *0.8 vs* baseline and *p* > *0.4 vs* baseline, respectively) (Table 2).

**Table 2:** Arterial stiffness indices and oxidative stress biomarkers.

	Vaping w/o nicotine			Vaping with nicotine			Sham vaping		
	BSL	After exposure	*p*	BSL	After exposure	*p*	BSL	After exposure	*p*
**Arterial stiffness indices**									
PWV (m.s^−1^)^*^	5.1 ± 0.1	5.1 ± 0.1	>*0.8*	4.9 ± 0.1	5.3 ± 0.1	***<0.0001***	5.1 ± 0.1	5 ± 0.1	>*0.4*
AIx75 (%)^*^	−3.8 ± 1.8	−4.5 ± 2.1	>*0.6*	−3.5 ± 1.5	1.9 ± 2.3	***0.013***	−2.7 ± 1.8	−4.3 ± 2	>*0.3*
AP (mm Hg)^*^	1.2 ± 0.6	0.5 ± 0.7	>*0.1*	1.2 ± 0.4	0.6 ± 0.8	>*0.2*	1.4 ± 0.6	1.4 ± 0.5	>*0.8*
Aortic PP (mm Hg)^*^	25.9 ± 1.3	26 ± 1.4	>*0.9*	25.6 ± 1.1	29.3 ± 0.9	***<0.0001***	26 ± 1.1	25.9 ± 1.5	>*0.8*
Aortic SBP (mm Hg)^*^	94.2 ± 1.6	96.6 ± 2.1	*0.05*	94.2 ± 1	103 ± 2.6	*0.004*	94.1 ± 1.4	93.1 ± 1.7	>*0.3*
SERV (%)^*^	194.8 ± 7.2	180.6 ± 7.2	*0.013*	193.4 ± 7	136.5 ± 6.7	***<0.0001***	187.5 ± 7.6	201 ± 7.6	***0.005***
*Oxidative stress biomarkers*									
Plasma MPO antigen (ng.mL^−1^)^†^	11.2 [8.2–16.6]	13.3 [9.8–16.3]	>*0.3*	13.6 [10–17.7]	18.9 [12.2–54.5]	***0.005***	11.3 [9.6–14.8]	11.3 [7.8–18.6]	>*0.4*
Plasma P-B-3-Cl-Tyr/Tyr ratio (10^−6^)^†^	59.8 [49–67.5]	57.1 [45.7–62.6]	*0.004*	55.7 [47.8–64.1]	55 [49.2–60.7]	>*0.2*	60.3 [48.2–71.9]	59.1 [48.6–67.1]	>*0.6*
Plasma Hcit/Lys ratio (10^−6^)^†^	218 [192.8–277.3]	220.5 [188–279.7]	*>0.5*	230.1 [195.5–316.6]	222.6 [183.3–376.1]	>*0.3*	223 [203–267]	215.7 [185.6–250.3]	>*0.5*

w/o denotes without; BSL, baseline; PWV, Pulse wave velocity; AIx75_;_ Augmentation index corrected for heart rate; AP, Aortic augmentation index; Aortic PP, Aortic pulse pressure; Aortic SBP, Aortic systolic blood pressure; SEVR, subendocardial viability ratio; MPO, Myeloperoxydase; P-B-3-Cl-Tyr/Tyr, Protein-bound-3-Chloro-thyrosine/Tyrosine ratio; Hcit/Lys: Homocitrulline/Lysine ratio.

^*^Mean ± SEM.

^†^Median [interquantile range].

#### Oxidative stress biomarkers and nicotine concentration assessments

Plasma myeloperoxidase and its oxidized proteins products: Compared to baseline, plasma myeloperoxidase increased after nicotine vaping exposure (13.6 ng. ml^−1^ [10–17.7] to 18.9 ng. ml^−1^ [12.2–54.4], *p* = *0.001*), but did not do so after vaping without nicotine or sham-vaping (*p* > *0.3* and *p* > *0.4*, respectively). In the nicotine vaping session, the Ach/SNP ratio was inversely correlated to the rise in myeloperoxidase in the plasma (r = −0.53, *p* = *0.017*). Vaping with nicotine and sham-vaping did not affect concentrations of serum protein-bound 3-chlorotyrosine/tyrosine ratio or homocitrulline/lysine ratio (All *p* > *0.2*). Compared to baseline, vaping without nicotine decreased protein-bound 3-chlorotyrosine/tyrosine ratio (x10^−6^) (59.8 [49–67.5] to 57.1 [45.7–62.6]; *p* = *0.004*) (Table 2).

Serum nicotine concentration: In the nicotinic vaping session, baseline median nicotine level was 0 ng. ml^−1^ [0–0] and rose to 8.9 ng.ml^−1^ [6.9–16.3] 30 minutes after vaping (*p* < *0.0001 vs*. baseline). Serum nicotine concentration did not correlate with any of the parameters we assessed, except with the total amount of nicotinic e-liquid vaped (r = 0.68, p = 0.001).

## Discussion

The main new findings of our study can be summarized as follow: in young and healthy tobacco smokers, acute exposure to high temperature vaporization of a pharmaceutical grade nicotine free PG/GLY mix (*50:50*): (1) did not alter microcirculatory function as well as arterial stiffness and oxidative stress; (2) whereas vaping the same mix plus nicotine decreased microcirculatory endothelial-dependent function, increased arterial stiffness, provoked a sustain raise in blood pressure and heart rate and increased plasma myeloperoxidase.

E-cigarettes are mainly used to quit smoking by former tobacco smokers who often suffer from cardiovascular disease^5,15^. The efficiency of nicotine replacement therapies is primarily determined by relief of smoking abstinence symptoms. Last generation high output wattage e-cigarettes, which vaporize large amount of e-liquid per puff, have been shown efficient to relieve these symptoms as they deliver quickly a high nicotine level^29,30^. Former smokers who completely shift to e-cigarettes purchase the last generation e-cigarettes we used in this study^12,13^, which make our results especially relevant for cardiovascular disease prevention. We are not aware of other studies which assess the acute cardiovascular effects of these new devices vaporizing e-liquid at high temperature. The cardiovascular parameters we measured have been shown very sensitive and reproducible to detect subtle changes in vascular regulation after acute exposure to various stimuli, and are commonly used to identify increased cardiovascular risk population^22,23,25,26^. The intense vaping conditions used in our study were intended to maximize our ability to detect any harmful effect of e-cigarette on microcirculation, arterial stiffness, hemodynamic parameters and oxidative stress. Overheated PG/GLY produces free radicals and volatile carbonyls (i.e. acrolein) by thermal degradation, which are potent vasoconstrictors and oxidative stressors^15,19–21^. When they are weaned from tobacco smoking, regular high wattage e-cigarettes users decrease nicotine concentration in the e-liquid, which in turn is vaped in larger amounts and thereby enhance volatile carbonyls emission^31^. The e-cigarette we used has been shown to emit these volatile carbonyls^9^, but using this device without nicotine under intense use conditions altered none of the above-mentioned parameters in our study.

Participants’ serum nicotine concentrations at 30 minutes after nicotine vaping were similar to those attained after tobacco cigarette smoking or last generation e-cigarettes vaping in other studies^14,32^. The symptomatic effects of nicotine are highly variable^33^ since all subjects achieved normal values of serum nicotine concentration after vaping, but four participants experimented nevertheless nicotinic symptoms (e.g. stomach ache, headache and weakness) and nine complained of some degree of throat irritation^34^. The sympathetic nervous system is activated when nicotine binds its cholinergic receptors, and this activates the cardiovascular system. The sympathomimetic effects of nicotine are mediated by the following mechanisms: activation of peripheral chemoreceptors, direct effects on the brain stem, intrapulmonary chemoreceptors stimulation, catecholamine release from the adrenals and direct release of catecholamines from vascular nerve endings. Among the numerous nicotine-released neurotransmitters, epinephrine, norepinephrine, dopamine, Ach, serotonin or also vasopressin could contribute to effects of nicotine on blood vessels^15^. We found that nicotine impaired Ach mediated vasodilation without disturbing the hyperemic test in response to heating after L-NAME or saline (placebo) pretreatment. Ach dilates vessels mainly by activation of NO synthase and prostaglandin (PG) production^35^. The heat-induced hyperemia in the presence of L-NAME assesses chiefly the contribution of the NO-mediated vasodilation in this response^22,23^. Thus, our results suggest that nicotine alters vasodilatation rather through a PG -dependent than a NO-dependent pathway. This is in agreement with studies where nicotine decreased PG synthesis in the vascular endothelium via an increase in oxidative stress^36–38^. Although not formally observed in our study, nicotine can also impair NO bioavailability^39^. Differences in the route and rate of nicotine administration, as well as the evidence that the effects of nicotine are highly variable in function of its pharmacokinetics, could perhaps explain these differences^18,33^. In addition, the variability in nicotine pharmacodynamics, and the limited number of subjects investigated in this study, could explain the lack of correlations between serum nicotine concentration and cardiovascular or oxidative stress parameters we assessed^18,33^.

We found that the oxidative effector enzyme myeloperoxidase increased in plasma early after nicotine vaping, but not in case of nicotine-free vaping. Myeloperoxidase is mainly stored in the azurophil granules of the polymorphonuclear neutrophils and monocytes^40^. Neutrophils are activated in the sputum of e-cigarettes users, but whether this can be attributed specifically to the effects of nicotine *per se* is unknown^41^. Whereas myeloperoxidase has a protective role in inflammatory processes, persistent generation of myeloperoxidase-derived oxidants may become deleterious and can also contribute to the development of cardiovascular diseases. Plasma myeloperoxidase levels are positively correlated with an increased cardiovascular risk^42^. The magnitude of microvascular endothelial dysfunction after nicotine vaping was related to the rise of plasma myeloperoxidase in our participants. *In vitro* nicotine exposure has been shown to activate leucocytes and promote release of myeloperoxidase acutely, and this impaired Ach mediated vasodilation^42^, as we also observed. Myeloperoxidase-induced endothelial dysfunction could be in part mediated by NO-independent mechanisms, an observation supported by the fact that NO-mediated vasodilation remained unaffected in our study. The other effects we observed on micro- and macro-circulatory functions, such as endothelial dysfunction and the rises in blood pressure, heart rate and arterial stiffness, are in good agreement with previous studies on the acute cardiovascular effects of nicotine^15,18^. Nicotine exerts pharmacologic effects that could contribute to acute cardiovascular events and accelerated atherogenesis experienced by cigarette smokers. Studies of nicotine medications indicate that the risks of nicotine without tobacco combustion are low compared to cigarette smoking, but are still of concern in people with cardiovascular disease^15,18,43^. Although PG/GLY vaporization under intense use conditions did not disturb cardiovascular function, this should however not motivate vapers to do so, since our results address only the effects of a single acute exposition on the cardiovascular system, while other important target organs such as the upper and lower airways were not assessed in this study.

We enrolled only occasional smokers with low cumulative cigarettes pack-year in this study, instead of never-smokers, because we believed that the latter would not be able to vape e-cigarettes as required in this protocol. Thus, our results may not necessarily apply to other groups such as heavy smokers and non-smokers. In addition, older subjects, with a higher cardiovascular risk or lung disease were not studied, and our results cannot be extrapolated to such conditions. Another limitation of this study is the small number of subjects investigated as well as the absence of their blinding during the exposure; but the latter is an intrinsic limitation of this kind of study. The participants were carefully selected and only those able to vape at high wattage were recruited, the majority of them reported to inhale the vapor directly to the lungs; which is not practiced by all the vapers. Twenty-five puffs lasting 4 seconds with more than 1.5 ml vaporized were investigated, this is quite different of regular vapers habits, who instead vape a total of 120–235 puffs a day during several sessions and consume a mean of 4 ml e-liquid per day^44^. Thus although our study resulted in a larger inhalation of e-liquid over a shorter period of time, there is no reason to believe that cardiovascular safety of PG/GLY inhalation in large amount we assessed would be more detrimental when used in lesser quantity. Last, regular vapers may not disclose the same response that the occasional smokers we investigated and this will require also further studies.

In conclusion, our study reveals the new finding that high temperature e-cigarette vehicles vaporization and inhalation in occasional smokers does not alter micro and macrovascular endothelial function, as well as oxidative stress. These effects are merely attributable to the nicotine present in the e-liquid.

## Electronic supplementary material

Supplementary File
