# Supplementary material for: Differential Effects of E-Cigarette on Microvascular Endothelial Function, Arterial Stiffness and Oxidative Stress: A Randomized Crossover Trial
Source: Sci Rep. 2018 Jul 10;8:10378. doi: 10.1038/s41598-018-28723-0 (PMC6039507; doi:10.1038/s41598-018-28723-0)
Supplement: Supplementary file 1 — Supplementary File [file 41598_2018_28723_MOESM1_ESM.pdf]

SUPPLEMENTAL MATERIAL

DIFFERENTIAL EFFECTS OF E-CIGARETTE ON MICROVASCULAR ENDOTHELIAL FUNCTION, ARTERIAL STIFFNESS AND OXIDATIVE STRESS: A RANDOMIZED CROSSOVER TRIAL

Martin Chaumont*[1,2], MD; Benjamin de Becker[1,2], MD; Wael Zaher[1,2]; Antoine Culié[1,2]; Guillaume Deprez[3]; Christian Melot[1,2]; Florence Reyé[4]; Pierre Van Antwerpen[4], PhD; Cédric Delporte[4], PhD; Nadia Debbas[5], MD, PhD; Karim Zouaoui Boudjeltia[6], PhD; and Philippe van de Borne[1,2], MD, PhD.

[1] Department of Cardiology, Erasme University Hospital, Université Libre de Bruxelles, Brussels, Belgium.

[2] Institute for Translational Research in Cardiovascular and Respiratory Sciences, Université Libre de Bruxelles, Brussels, Belgium.

[3] Department of Clinical Chemistry, Université Libre de Bruxelles, Brussels, Belgium.

[4] Laboratory of Pharmaceutical Chemistry, Faculty of Pharmacy, Université Libre de Bruxelles, Brussels, Belgium.

[5] Department of Cardiology, Saint-Pierre Hospital, Université Libre de Bruxelles, Brussels, Belgium.

[6] Laboratory of Experimental Medicine (ULB 222 Unit), CHU de Charleroi, A. Vésale Hospital, Université Libre de Bruxelles, Montigny-le-Tilleul, Belgium.

1. Participants

All participants were recruited through advertisements at Université Libre de Bruxelles at our academic center (Erasme University Hospital, Brussels, Belgium) between January 2017 and November 2017. They were: i) occasional smokers of tobacco cigarettes (less than 20 tobacco cigarettes per week); ii) not using any other forms of tobacco or nicotine replacement products; and iii) not abusing of substances. All participants undertook one screening visit and were randomly assigned to three experimental sessions. During the screening visit, a full medical history and a physical examination were obtained. Subjects were defined healthy on the basis of: i) no history of acute or chronic disease; ii) no history of cardiovascular symptoms; iii) normal blood parameters; iv) no intake of antioxidant compounds or other medication except oral contraceptive; and v) blood pressure and heart rhythm within the normal limits. All the subjects were trained during the screening visit to inhale vapor from high wattage electronic cigarette as per protocol. Staff members gave advices on vaping especially about lips overheating, which could be easily avoided. Participants were instructed to notify to the investigators any feeling of throat irritation ("dry-hit").

2. Study design

This randomized study was placebo-controlled, single-blind with a three-period crossover design. The 3 experimental periods were separated by a minimum of seven days of wash-out to minimize carryover effects. Vaping (with and without) or sham-vaping (placebo) were followed by a 3-h observation period. Measurements were done continuously before exposure (vaping with and without nicotine and sham-vaping) and then through all the

experimental session. The sequence order was randomized. Twenty-five participants were randomized to three groups (group 1: sham-vaping first followed by vaping without nicotine followed by vaping with nicotine; group 2: vaping without nicotine first followed by sham-vaping followed by vaping with nicotine; group 3: vaping with nicotine first followed by sham-vaping followed by vaping without nicotine). Three sessions were completed by 21 subjects, whereas 2 of them performed only two sessions (one was not reachable for the third session and one participant refused to complete the study without giving any reason) and 2 other participants performed only one session (one was not reachable for the second session and the other refused to participate to the second session because of vein puncture). The measurements were carried out after 20 minutes of rest in a quiet room with the temperature maintained constant at 23±1°C in the supine position. Speaking and sleeping were not allowed during all the experimental sessions. For each subject, the three study sessions were performed at the same time of the day to minimize circadian variations of skin temperature. Before each experimental session, participants were negative for urine cotinine (urine dipstick - semi-quantitative detection threshold of 600 $ng.ml^{-1}$ - NarcoCheck©, Paris, France), negative for urine tetrahydrocannabinol (urine dipstick - semi-quantitative detection threshold of 50 $ng.ml^{-1}$ - NarcoCheck©, Paris, France) and had fractional exhaled carbon monoxide (SineFuma©, Breda, Holland) level less than 5 ppm. [1] All the participants abstained from caffeine- and alcohol-containing drinks for 48 hours, and had to refrain from smoking for at least 48h before each study period. Subjects were asked to fast at least 12h prior to experimentation, but water ingestion was permitted to promote euhydration. The volunteers did not wash their arms and forearms on the morning of the experiment. They were excluded from the experiment if they had used nonsteroidal anti-inflammatory drugs ≤ 3 days before

each session. Participants were asked to abstain from physical exercise for 48h before the sessions to avoid the short-term impact of exercise on endothelial function.

3. Vaping protocol

The e-liquids were prepared by the department of Pharmacy of the Erasme hospital and consisted of 50% propylene glycol (PG) and 50% vegetable glycerin (GLY) (pharmaceutical grade - Fagron©, Waregem, Belgium). One e-liquid was without nicotine (0 mg/ml), whereas nicotine (Nicobrand©, Coleraine, UK) was added in the other (3 mg/ml). PG and GLY are carriers which allow the vaporization process and are often found in commercial e-liquids in a *50:50* mix, [2] as in our study. We excluded flavors in order to study the impact of PG/GLY vaporization and nicotine *per se* on outcomes parameters. The vaping device (purchased in December 2016 from a local store) was an Alien 220 box mod and a TFV8 baby beast tank (Smoke©, Shenzen, China). We used the MXJO (Mxjotech©, Shenzen, China) IMR 18650 3000 mAh 35A variable voltage/variable wattage batteries. The clearomizer contained a replaceable bottom heating dual Kanthal coil (V8 Baby-Q2 Core (0.4Ω dual coils) -Smoke©, Shenzen, China). Air inflow position was systematically opened to the maximum. For the purpose of this study, e-cigarettes were set-up at 60 Watts (wattage mode). Products were prepared according to the manufacturer’s instructions. Vaping devices were entirely cleaned, fully charged and fully filled with liquid before each session. The V8 Baby-Q2 Core was replaced after each of two-vaping sessions. The period of exposure was supervised by an unblinded member of the team. The blinded experimenters did not prepare the device nor supervise the exposure. Each vaping session consisted of 25 puffs (4-s puffs at 30-s intervals). At the end of each puff, the unblinded member verified: 1) the lack of vapor in the subject

mouth to prevent superficial vaping and 2) the presence of exhaled vapor. After the vaping, the subjects were questioned about symptoms suggestive of dry hit. The e-cigarette was weighed before and after the session to determine the exact amount of liquid vaporized (vaping sessions) or leaked (sham-vaping).

4. Indices of skin microcirculatory blood flow

Acetylcholine and sodium nipride iontophoresis: we used a MOORLDI2-IR laser Doppler imager (Moor Instruments©, Axminster, UK) to assess cutaneous microcirculatory flow after vaping and sham-vaping. The cutaneous microcirculatory blood flow was measured in a surface of 3.8 cm^2 on the ventral side of the right forearm, as described previously. [3] At the end of vaping sessions or sham-vaping, 12 scans were acquired. The 2 first scans corresponded to the baseline cutaneous blood flow. The 10 following scans were performed concomitantly to iontophoresis, which was used during 26 minutes to administer percutaneously acetylcholine (2.5 ml in the anode chamber) and sodium nitroprusside (2.5 ml in the cathode chamber). The concentrations of acetylcholine and sodium nitroprusside solutions were 2 g/100 ml in deionized water. The Plexiglas iontophoresis chambers (ION 6; Moor Instruments©, Axminster, UK) were connected to the anode and cathode with an iontophoresis controller (MIC 2, Moor Instruments©, Axminster, UK), which applied a current of 100 µA. The unspecific vasodilation response to galvanic iontophoretic current was avoided by application of 5% EMLA cream (lidocaine 2.5% and prilocaine 2.5%; AstraZeneca©, London, UK) one hour before the test. [3, 4] Avoiding superficial vessels and body hair, the iontophoresis chambers were placed at the same location for each of the two sessions on the basis of multiple pictures taken during the experimental sessions.

Heat test in presence of L-N-arginine-methyl-ester iontophoresis: as previously described,[3, 4] we tested on the contralateral arm the cutaneous hyperemic response to local heating after pretreatment with iontophoresis of L-*N*-arginine-methyl-ester, to assess vasodilation mediated by nitric oxide. As for acetylcholine and sodium nitroprusside iontophoresis, 5% EMLA cream was applied to the skin surface of forearm ventral side. The concentration of the L-*N*-arginine-methyl-ester solution was 20 mM in deionized water. The anode and the cathode chambers were filled with 2.5 ml of L-*N*-arginine-methyl-ester and NaCl 0.9 g/100 ml solution (Baxter), respectively. The cathode chamber with the NaCl 0.9 g/100 ml solution (Baxter) served as control electrode. After iontophoresis of L-*N*-arginine-methyl-ester and NaCl 0.9 g/100 ml solutions, a total of 12 scans was obtained. After the 2 first scans (baseline cutaneous microcirculatory blood flow), skin heater electrodes connected to a temperature monitor (SH02, Moor Instruments©, Axminster, UK) heated the skin up to 44°C for 26 minutes with the view to achieve maximal skin vasodilation. Acetylcholine, sodium nitroprusside and L-*N*-arginine-methyl-ester solutions were stored in Eppendorfs at -20°C, and thawed one hour before the start of the experiment.[3, 4]

5. Haemodynamic measurements

After 20 minutes of comfortable rest, in the supine position, humeral blood pressure was determined (Mercury sphygmomanometer, WelchAllyn©, New York, USA) according to guidelines.[5] Blood pressure measurements were performed before (baseline) and immediately after vaping sessions or sham-vaping. A cuff was placed on the middle phalanx of the right middle finger in order to obtain a finger blood pressure waveform with a beat-to-beat hemodynamic monitoring system (Finometer Pro©, FMS, Amsterdam, the Netherlands).

A generalized transfer function reconstructed simultaneously humeral blood pressure waveforms. [6] This permitted a continuous monitoring of the humeral systolic and diastolic blood pressures throughout all the experimental sessions duration. Pulse rate was monitored throughout the study by the finometer recordings.

6. Arterial stiffness assessment

6.1. Aortic wave reflection assessment

Using applanation tonometry at the level of left radial pulse, arterial waveforms were recorded during 8-second with a high-fidelity SPC-301 micromanometer (Millar Instrument©, Texas, USA) by means of a fully automated and validated system (SphygmoCor, Atcor Medical), version 9.0 software (AtCor Medical©, New South Wales, Australia). Three minutes before aortic wave reflection assessments, manual blood pressure measurements (Mercury sphygmomanometer, WelchAllyn©, New York, USA) were performed according to guidelines [5] in order to calibrate radial pulse wave. Aortic pressure waveforms were derived from the peripheral radial pressure waves using a validated generalized transfer function. [7] This has been shown to accurately estimate aortic pressure waveforms based on the assumption that mean blood pressure does not change along the arterial tree. In case of decreased aortic compliance or increased peripheral resistance, the reflected wave come back faster to the aorta. This can be assessed by the augmentation index (AIx; 100× augmentation pressure/pulse pressure), representing the pressure boost induced by the return of these reflected waves to the aorta, expressed as a percentage of the pulse pressure. Higher values of AIx indicate an earlier return of the reflected wave to the aorta. Ventricular ejection time

determines the aortic pressure waveform. In case of heart rate alterations with ventricular ejection time modifications, AIx changes may be masked or overestimated. This is why all the AIx presented in the article are corrected for heart rate (Aix_{75}) according to the linear relationship established by Wilkinson et al, [7] namely, that for every 10-bpm increase in heart rythm, AIx decreases by 4%.

6.2. Pulse wave velocity measurements

We assessed the carotid-femoral pulse wave velocity (PWV) using a sequential waveform measurements approach at carotid and femoral sites with applanation tonometry (SPC-301 micromanometer) and SphygmoCor software as previously described. [9] The pulse waves time period between the carotid and femoral sites was assessed with an electrocardiograph-derived R wave as a fixed point. This pulse wave time was the average of 10 consecutive beats. The difference between the path in cm from the left carotid sampling site to the suprasternal notch, and the path in cm from the left femoral sampling site to the suprasternal notch was used to define the distance which the pulse wave travels.

7. Oxidative stress biomarkers analysis and nicotine assessment in blood

Blood was drawn in the left antecubital vein just before, as well as 30 minutes after exposure, and immediately centrifuged at 3500 g for 10 minutes to obtain the supernatant, which was aliquoted. Serum and plasma samples were frozen and stored at -80 °C immediately after centrifugation and aliquoting.

Plasma total myeloperoxidase, protein-bound 3-chlorotyrosine and homocitrulline: Total myeloperoxidase plasma content was measured using a sandwich human myeloperoxidase ELISA kit (Quantikine ELISA Kit, R&D Systems©, Abingdon, United Kingdom). [10] Protein-bound 3-chlorotyrosine and homocitrulline, two reaction products of protein damage due to myeloperoxidase activity, were analyzed in plasma proteins by means of a total hydrolysis method followed by a liquid chromatography mass spectrometry analysis (Agilent Technologies©, Santa Clara, CA, USA) as previously described by our team. [11] These analyses were performed at the Campus de la Plaine, Therapeutic Chemistry, Faculty of Pharmacy, Université Libre de Bruxelles.

Serum Nicotine assessment: Nicotine was assessed in the serum before and 30 minutes after the intervention by means of a mass spectrometer (Agilent QQQ 6490, Agilent©, Santa Clara, USA) with a jet stream electrospray ion source. An Agilent 1260 series LC system was used for quantification of plasma nicotine. Samples were first extracted on Oasis MCX SPE columns (Waters©, Massachusetts, USA) according to the manufacturer's recommendations. Serum samples were spiked with deuterated nicotine internal standard and acidified with phosphoric acid 4% then centrifuged for 5 min at 5000 rpm. SPE cartridges were conditioned with Methanol and water. Samples were then transferred to the extraction columns. The cartridges were then washed with acidified water (2% formic acid) and methanol. The nicotine was eluted with methanol (2% ammonia), evaporated to dryness at room temperature under nitrogen and reconstituted with a mix of water and acetonitrile. LC separation was then performed on an Agilent HILLIC phase column (100 mm × 2.1 mm i.d., 2.7 µm particle size) maintained at 45°C with an isocratic mode at a flow rate of 0.4 ml/min. The autosampler tray

at 10°C and the injection volume was 2 µL. Mobile phase was a mix of 50mmol/L aqueous ammonium formate with 0.1% formic acid and acetonitrile. The mass spectrometer was operated in positive ESI mode. Nitrogen was used as nebulizer, turbo (heater) gas, curtain, and collision-activated dissociation gas. The capillary voltage was +3050V. The ion source gas temperature was 170°C and jet stream gas temperatures 400°C with flows of 12 L/min. MassHunter software (Agilent©) was used for system control, data acquisition, and data processing.

8. Data analysis

All the measurements detailed thereafter were analyzed in a blinded fashion.

*Cutaneous microvascular blood flow measurements (*MOORLDI2-IR©*):* the final analysis included 21 participants (21/25) for the comparisons between vaping with and without nicotine and sham-vaping (two participants performed only one session, two other only two sessions). As in our previous studies, [3, 4] skin blood flow was measured automatically (LDI version 5.3D software, Moor Instruments©, Axminster, UK) and was expressed in Perfusion Units, PU (arbitrary units of blood flow). The skin blood flow values during hyperemia tests were expressed as raw data minus the mean of the two baseline scans. The response to stimulation was quantified as the absolute unit change of every scan minus the baseline skin blood flow value. The area under the curve (AUC) was calculated by summing each of the 10 measurements of skin vasodilation in response to hyperemic tests. We analyzed the effect of L-NAME iontophoresis in both vaping and sham-vaping conditions. As previously, particular

attention was paid on the effect of these interventions on the late phase of the skin reaction to heating. [3, 4] The late phase AUC during heating-induced vasodilation was defined as the AUC observed after the initial peak of the hyperemic reaction, i.e., after the fifth scan.

Hemodynamic measurements (Finometer Pro©): the final analysis included 20 participants (20/25) for the comparisons between vaping with and without and sham-vaping (two participants performed only one session, two other only two sessions and one had a corrupted Finometer data file). Finometer values were recorded and analyzed off-line (Beatscope© 1.1a, FMS©, Amsterdam, the Netherlands). One value was taken to summarize the baseline period and vaping exposure, respectively. Thereafter, we followed hemodynamic parameters during 120 minutes. These measurements were averaged during 4 successive periods of continuous recording lasting 30 minutes each. Finometer hemodynamic values were corrected for humeral blood pressure values.

Aortic wave reflection assessments (Sphygmocor©): in the sham-vaping session, the final analysis included 23 participants (23/25) for the comparison between baseline and 30 minutes post sham-vaping (one participant did not perform the sham-vaping session; in one other participant, Sphygmocor quality criteria was not achieved). In the nicotine free vaping session, the final analysis included 23 participants (23/25) for the comparison between baseline and 30 minutes post nicotine free vaping (one participants did not perform the nicotine free vaping session and for one other participant we did not reach Sphygmocor quality criteria). In the nicotine vaping session, the final analysis included 21 participants (21/25) for the comparison between baseline and 30 minutes post nicotine vaping (four participants did not perform the nicotine vaping session). At least two AIx75 measurements were performed, and their mean

value was calculated. If the 2 measures differed by >3%, the mean of three measurements was computed, according to the Task Force III recommendations for user procedures. Recordings where the systolic or diastolic variability of consecutive waveforms exceeded 5% or the amplitude of the pulse wave signal was less than 80 mV were discarded. [12, 13] These measurements were made ten minutes before and five-minute after exposure.

Pulse wave velocity assessment (Sphygmocor©): in the sham-vaping session, the final analysis included 24 participants (24/25) for the comparison between baseline and 30 minutes post sham-vaping (one participant did not perform the sham-vaping session). In the nicotine free vaping session, the final analysis included 23 participants (23/25) for the comparison between baseline and 30 minutes post nicotine free vaping (one participants did not perform the nicotine free vaping session and for one other participant we did not reach Sphygmocor quality criteria). In the nicotine vaping session, the final analysis included 21 participants (21/25) for the comparison between baseline and 30 minutes post nicotine vaping (four participants did not perform the nicotine vaping session). The following SphygmoCor quality criteria were monitored: 1) pulse waveforms consistent in size and shape; 2) standard deviation for carotid and femoral sites <6% of the mean wave propagation time; and 3) PWV standard deviation <10% of the mean. Borderline readings with PWV standard deviation between 10% and 15% were retained only if repeated PWV measurements were consistently in this range. The mean of two PWV measurement were taken. If these differ by more than 0.5 m/s, the median of three measurement was taken. [14] PWV measurements were performed immediately before and ten-minutes after exposure.

Plasma myeloperoxidase: in the sham-vaping session, the final analysis included 21 participants (21/25) for the comparison between baseline and 30 minutes post sham-vaping

(one participants did not perform the sham-vaping session; in one other participant, we had not enough serum to perform analysis; in two other participants, we could not achieve a satisfactory vein puncture). In the nicotine free vaping session, the final analysis included 22 participants (22/25) for the comparison between baseline and 30 minutes post nicotine free vaping (one participant did not perform the nicotine free vaping session and in two other participant we had not enough serum to perform analysis). In the nicotine vaping session, the final analysis included 20 participants (20/25) for the comparison between baseline and 30 minutes post nicotine vaping (four participants did not perform the nicotine vaping session; in one other participant, we could not achieve a satisfactory vein puncture).

Protein-bound 3-chlorotyrosine and homocitrulline analysis: in the sham-vaping session, the final analysis included 22 participants (22/25) for the comparison between baseline and 30 minutes post sham-vaping (one participants did not perform the sham-vaping session; in two other participants, we could not achieve a satisfactory vein puncture). In the nicotine free vaping session, the final analysis included 24 participants (24/25) for the comparison between baseline and 30 minutes post nicotine free vaping (one participant did not perform the nicotine free vaping session). In the nicotine vaping session, the final analysis included 20 participants (20/25) for the comparison between baseline and 30 minutes post nicotine vaping (four participants did not perform the nicotine vaping session; in one other participant, we could not achieve a satisfactory vein puncture).

9. Sub-ohm vaping online survey

Sub-ohm vaping consists of using e-cigarettes delivering high energy level to low coil resistance. Despite sub-ohm vaping seems increasingly popular, there is no data about this practice. In order to better characterize sub-ohm vaping conditions in regular users, we launched during the ten last days of November 2017 an online survey on the forum “UBV – BDB – Union Belge Pour La Vape / Belgische Damp Bond”. This forum has more than 3700 Belgian members defending vaping interests, which are its primary purpose. The survey collected information about habitual user consumption habits (PG/GLY proportion, wattage level, coil resistance value, mean e-liquid quantity consumed per day, nicotine strength in the e-liquid) and reasons for sub-om vaping. We collected data from 1152 responders, who completed the questionnaire. The majority of responders were male (81%), 61% were between 18 and 40-year-old and 37% were older than 40-year, 31.2% vaped since one to two years; and half of them (51%) vaped since at least two years. A mix PG/GLY (50:50) was used by 34% and the majority vaped a mix of PG/GLY with more GLY (44.8%). Sixty percent vaped with nicotine concentration comprised between 1 and 3 mg/ml and ten percent vaped without nicotine. The most often reported wattage range used was between 20 and 40 W (28%), followed by 40 and 60 W (27.6%). Thirty-four percent vaped at a higher wattage than 60 W. Resistance lower than 0.5 ohm was the most frequently used (64%), followed by resistance value between 0.5 and 1 Ohm (27.2%). Whereas 40% vaped more than 10 ml e-liquid per day, forty-eight % vaped between 4 and 10 ml per day. Reasons reported to vape at high wattage were to increase vapor (65%) and heat (22%) production and to enhance nicotine

throat hit (24%). These results confirm that the e-cigarette exposition we used in our study is clinically relevant in the vaping community.

10. Flow diagram – CONSORT

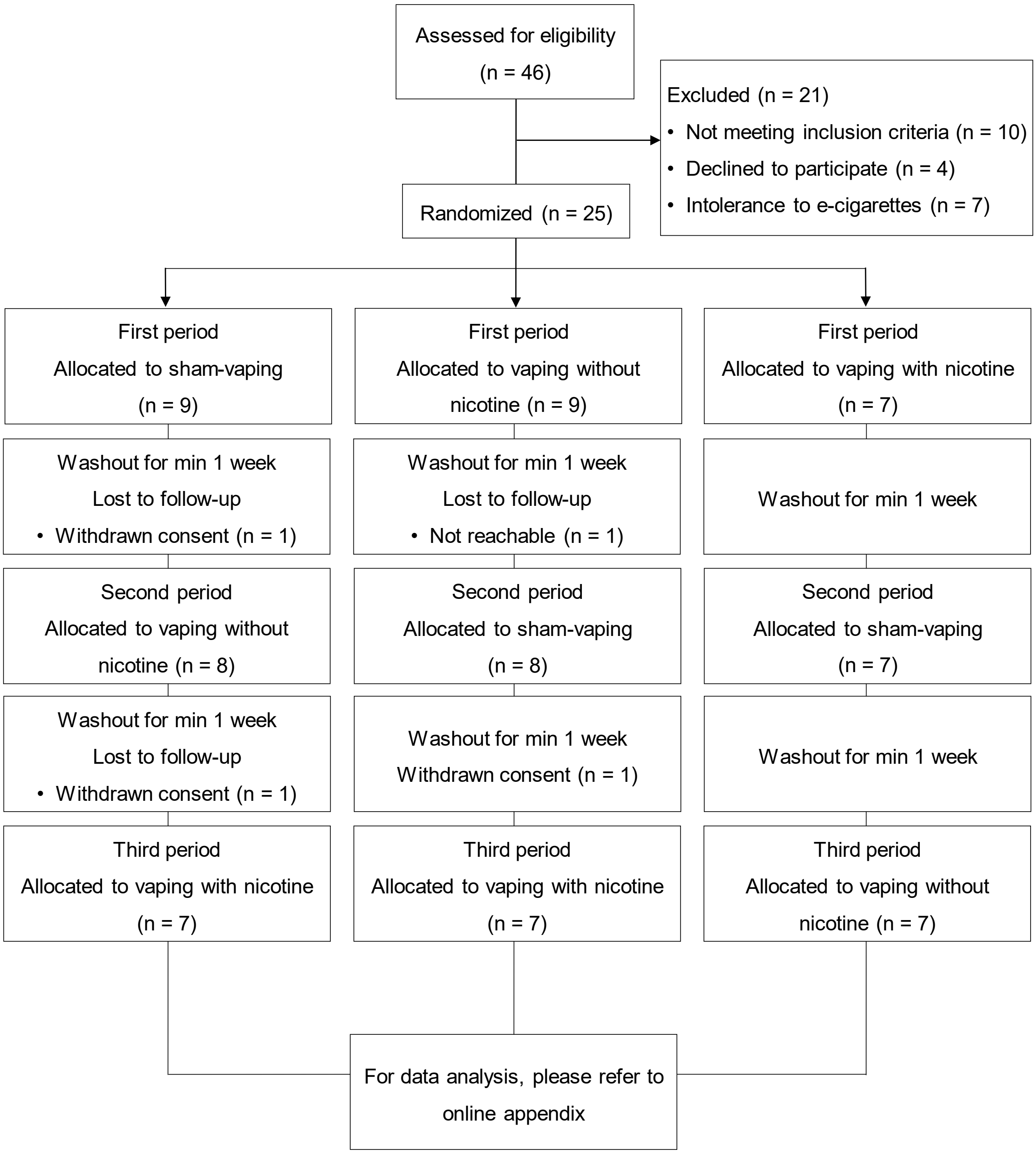

Flow diagram of the participants course during the study (CONSORT)

References

1. Ferrari, M. et al. Short-term effects of a nicotine-free e-cigarette compared to a traditional cigarette in smokers and non-smokers. *BMC Pulm Med*. **15,** 120 (2015).

2. Chen, Z., Zeng, D.D. Mining online e-liquid reviews for opinion polarities about e-liquid features. *BMC Public Health.* 17, 633 (2017).

3. Dreyfuss, C. et al. L-NAME iontophoresis: a tool to assess NO-mediated vasoreactivity during thermal hyperemic vasodilation in humans. *J Cardiovasc Pharmacol.* **61,** 361-8 (2013).

4. Wauters, A. et al. Acute exposure to diesel exhaust impairs nitric oxide-mediated endothelial vasomotor function by increasing endothelial oxidative stress. *Hypertension*. **62,** 352-8 (2013).

5. Pickering, T.G. et al; Subcommittee of Professional and Public Education of the American Heart Association Council on High Blood Pressure Research. Recommendations for blood pressure measurement in humans and experimental animals: Part 1: blood pressure measurement in humans: a statement for professionals from the Subcommittee of Professional and Public Education of the American Heart Association Council on High Blood Pressure Research. *Hypertension*. **45,** 142-61 (2005).

6. Guelen, I. et al. Finometer, finger pressure measurements with the possibility to reconstruct brachial pressure. *Blood Press Monit*. **8,** 27-30 (2003).

7. Karamanoglu M, O'Rourke MF, Avolio AP, Kelly RP. An analysis of the relationship between central aortic and peripheral upper limb pressure waves in man. *Eur. Heart J*. **14,** 160–7 (1993).

8. Wilkinson, I.B., MacCallum, H., Flint, L., Cockcroft, J.R., Newby, D.E., Webb, D.J. The influence of heart rate on augmentation index and central arterial pressure in humans. *J. Physiol.* **525,** 263–70 (2000).

9. Butlin, M., Qasem, A. Large Artery Stiffness Assessment Using SphygmoCor Technology. *Pulse (Basel).* **4,** 180-192 (2017).

10. Franck, T. et al. A new easy method for specific measurement of active myeloperoxidase in human biological fluids and tissue extracts. *Talanta*. **80,** 723-9 (2009).

11. Delporte, C. et al. Simultaneous measurement of protein-bound 3-chlorotyrosine and homocitrulline by LC-MS/MS after hydrolysis assisted by microwave: application to the study of myeloperoxidase activity during hemodialysis. *Talanta*. **99,** 603-9 (2012).

12. Van Bortel, L.M. et al. Clinical applications of arterial stiffness, Task Force III: recommendations for user procedures. *Am J Hypertens*; **15,** 445-52 (2002).

13. Adamopoulos, D., Argacha, J.F., Gujic, M., Preumont, N., Degaute, J.P., van de Borne, P. Acute effects of nicotine on arterial stiffness and wave reflection in healthy young non-smokers. *Clin Exp Pharmacol Physiol.* **36,** 784-9 (2009).

14. Van Bortel, L.M. et al; Artery Society; European Society of Hypertension Working Group on Vascular Structure and Function; European Network for Noninvasive Investigation of Large Arteries. Expert consensus document on the measurement of aortic stiffness in daily practice using carotid-femoral pulse wave velocity. *J Hypertens*. **30,** 445-8 (2012).
